# Medical Brain Drain From Southeastern Europe: Using Digital Demography to Forecast Health Worker Emigration

**DOI:** 10.2196/30831

**Published:** 2021-11-30

**Authors:** Tado Jurić

**Affiliations:** 1 Catholic University of Croatia Zagreb Croatia

**Keywords:** digital demography, Google Trends, the emigration of doctors and nurses, medical brain drain, Croatia, demography, brain drain, emigration, doctors, nurses, Western Balkans, health care workers, health professionals, health systems, jobs, Germany, personnel, migration, workforce, medical professionals

## Abstract

**Background:**

This paper shows that the tools of digital demography, such as Google Trends, can be used for determining, estimating, and predicting the migration of health care workers (HWs), in this case, from Croatia and the Western Balkans (WB) to Germany and Austria.

**Objective:**

This study aims to test the usefulness of Google Trends indexes to forecast HW migration from Croatia and the WB to Germany and Austria. The paper analyzes recent trends in HW mobility in Europe and focuses specifically on mobility patterns among medical doctors and nurses using digital demography. Without increased emigration in the last 10 years, Croatia and the WB would have 50% more HWs today, and this staff is now crucial in the fight against a pandemic. Furthermore, the COVID-19 pandemic contributed to the increase in emigration.

**Methods:**

A particular problem in analyzing the emigration of HCWs from Croatia and the WB is that there is no system for monitoring this process. Official data is up to 3 years late and exists only for persons deregistered from the state system. Furthermore, during the pandemic, the “normal” ways of data collection are simply too slow. The primary methodological concept of our approach is to monitor the digital trace of language searches with the Google Trends analytical tool. To standardize the data, we requested the data from January 2010 to December 2020 and divided the keyword frequency for each migration-related query. We compared this search frequency index with official statistics to prove the significance of the results and correlations, and test the model’s predictive potential.

**Results:**

All tested migration-related search queries, which indicate HCWs’ emigration planning, showed a positive linear association between Google index and data from official statistics (Organisation for Economic Co-operation and Development: Serbia *R*^2^=0.3381, Bosnia and Herzegovina [B&H] *R*^2^=0.2722, Croatia *R*^2^=0.4515). Migration-related search activities such as “job application + nurses” from Croatia correlate strongly with official German data for emigrated HWs from Croatia, Serbia, and B&H. Decreases in Google searches were correlated with the decrease in the emigration of HWs. Thus, this method allows reliable forecasts for the future.

**Conclusions:**

This paper highlights that the World Health Organization’s list of countries with HWs shortages should be updated to include Croatia and the countries from the WB. The issue of the European Union drawing HWs from the EU periphery (Croatia) and nearby countries (B&H, Serbia) clearly shows a clash between the EU freedom of movement and the right to health care and a need to ensure a health care workforce in all European regions. Understanding why HWs emigrate from Croatia and the WB, and the consequences of this process are crucial to enabling state agencies and governments to develop optimal intervention strategies to retain medical staff. The benefit of this method is reliable estimates that can enable a better response to a possible shortage of HWs and protect the functioning of the health system. The freedom of movement of workers in the EU must be supplemented with a common pension and health care system in the EU.

## Introduction

### Background

The health care system across Europe faces demographic ageing of both staff and users, increasing the demand for health needs and care. On the one hand, the demand and, on the other hand, the supply and low wages of medical labor are shaping the current situation in the European Union. One-third of the EU 27 members are affected by the shortage of nurses, and one-half of the EU 28 countries report shortages of medical doctors [1]. The COVID-19 emergency has again confirmed that all EU countries have weaknesses in their health system, and one of them is the inadequate supply of health workers [2]. Developed EU countries are tackling the shortage by immigrating health workers, mainly from Croatia and the Western Balkans (WB). At the same time, in the countries from which health workers emigrate, negative consequences are felt, which is even more pronounced during the pandemic. In addition, the pandemic will likely increase global competition for doctors [3] and nurses.

European Observatory books on health workers’ mobility [4] showed in 2015 that there are gaps in understanding the magnitude of health professional mobility, particularly concerning EU enlargement. “There is also no overview of the motivators that drive the mobile European health workforce...or any systematic mapping of the impacts of health professional mobility.” In addition, it is emphasized that knowledge on country responses to health professional mobility in the European Union is limited [4]. Despite this, there were no significant developments [2]. The World Health Organization (WHO) report [5] about countries with critical health workforce shortages does not even state that these issues are also relevant in European countries and that the list should be updated to include the countries Bosnia and Herzegovina (B&H), Serbia, and Croatia.

### A New Approach in the Field of Health Care Worker Migration

A particular problem in the analysis of the emigration of health care workers (HWs) from Croatia and the WB is that there is no system for monitoring this process. Official data is up to 3 years late and exists only for persons who have deregistered or, in the case of non-EU countries, requested a work visa as a medical professional. However, measuring HW mobility is a challenge for most countries, and therefore, new policy instruments and coordination at the EU level and new methods of monitoring trends in the movement of medical staff should be introduced. This study shows that the analytical tool Google Trends (GT) can give useful complement data to the knowledge of demography, especially in the field of HW migration in the European Union.

After briefly showing the results of relevant studies in the next section, we will focus on the emigration of health care professionals from Croatia and the WB to Germany and Austria, and the results we gained with the approach of digital demography. We used Croatia, Serbia, and B&H as a case study because this region is, according to the United Nations (UN), demographically one of the most affected regions due to depopulation in the world [6] and a region with a critical shortage of medical staff. In addition, there are no studies of this type (digital demography) in Croatia and the broader region of Southeast Europe. The primary hypothesis of this paper is that the analytical tool GT is a valuable source of data for determining, estimating, and predicting migrations of HCWs.

### Emigration of Health Workers From Croatia and the Western Balkans to Germany and Austria

#### Methodological Confusion in Data Collection About the Migration of Health Workers

Literature on the emigration of health workers from the countries of the WB and Croatia is scarce. Particularly useful contributions specific to the WB are the works of Mara [2,7], while the works on a broader topic come from the authors Adovor et al [8], Britnell [9], and The European Observatory on Health Systems and Policies [10]. Zeisler et al [11] presented an engaging innovative methodological approach to data collection (albeit with classical methods). However, none of the papers managed to resolve the methodological confusion in data collection about the migration of health workers or estimate the exact number of emigrated nurses and doctors from Croatia and the WB. In all three analyzed countries (B&H, Serbia, and Croatia), no single authority documents health professional mobility. Therefore, this required a more comprehensive search for documentation in international sources. Official data from Eurostat, UN, Organisation for Economic Co-operation and Development (OECD), Bundesamt für Migration und Flüchtlinge, and DESTATIS were used to estimate the number of medical staff from B&H, Serbia, and Croatia who emigrated to EU countries, while digital demography was used to model future trends.

A fundamental limitation in the preparation of the paper is the shortage of official data from domestic institutions on the number of emigrated health workers. A particular problem in demographic research in Southeast Europe is the methodological inconsistency and questionability of official data at several levels (unpublished study by Jurić). Another problem is the impossibility of comparing data identically for Croatia, B&H, and Serbia due to the lack of the same methodology in collecting data. Croatian doctors and nurses as citizens of the European Union can freely migrate and work in Germany and Austria, but doctors and nurses from Serbia or B&H need visas.

#### Emigration From Croatia, Serbia, and B&H Since 2013

Since it became a member of the European Union in 2013, an average of 50,000 people emigrate from Croatia every year, most often to Germany (85% of all emigrants) [12]. In the first half of 2019, Croatia, along with Bulgaria, had the highest percentage of emigration of all EU members [13].

Data for B&H shows that from 2014 to 2018, a net reduction in the workforce, primarily due to emigration, was 113,000 workers, or 10% of the total workforce (unpublished study by Jurić). According to Eurostat data [14] in the period 2014-2019, the number of citizens from B&H who were granted the first permit by any reason in the European Union was 226,519. The structure by country shows that most people from B&H are emigrating to Germany (35.68%), Slovenia (18.40%), Croatia (18.13%), and Austria (10.00%). The structure of issued permits shows that, since 2016, the dominant reason for emigration is to work in another country (58.26% in 2019) (unpublished study by Jurić).

According to OECD, in the period 2014-2019, the population of Serbia decreased by 187,688. Like in B&H, most permits are issued to citizens in the category 25-45 (unpublished study by Jurić). Of this number, 60.09% emigrated to Germany, 14.16% to Austria, and 4.94% to Slovenia [15]. Eurostat data for the period 2014-2019 showed that the European Union issued 248,759 first permits for any reason for Serbian citizens [16], which is 3.59% of the total population of Serbia. Of this number, 100,315 or 40.3% were issued for work purposes (unpublished study by Jurić).

### Nurses From Croatia and the Western Balkans in Germany

According to the German Federal Employment Service, in 2019, over 65,000 citizens of Croatia and the WB work in Germany’s health and care sector. According to estimates [17], Germany imports more than 10,000 caregivers and nurses from Croatia and the WB every year, mainly from B&H and Serbia but also from Macedonia, Kosovo, etc [18]. In 2017, of the 4600 foreign-trained nurses who moved to Germany, close to 32% originated from WB countries [2] (Table 1).

**Table 1:** Number of nurses from Croatia and the Western Balkans in Germany in 2018 and 2019 (n=57,288 from the Western Balkans and n=7500 from Croatia).^a^

Year	B&H^b^, n	Serbia, n	Kosovo, n	Albania, n	North Macedonia, n	Montenegro, n
2018	19,631	13,598	7204	5206	4094	993
2019	21,789	15,216	8050	6642	4487	1104

^a^Source: Own elaboration from Statistik der Bundesagentur für Arbeit [19].

^b^B&H: Bosnia and Herzegovina.

Abeitsagentur does not give data for Croatia, but OECD shows that in the period from 2011 to 2019, 1283 nurses emigrated from Croatia to Germany, while the Croatian chamber shows 7500. When we put these data of 65,288 emigrated HWs from this region in context, we can see that this is a higher number than the total number of nurses in Croatia and B&H together. Without such intense emigration in the last 10 years, the regions of Croatia and the WB would have 50% more HCWs today. It is necessary to emphasize that this staff is crucial in the fight against a pandemic.

Concerning the trained staff, the numbers become even more alarming. In Croatia, for example, in the last 5 years, an average of 5100 nurses have completed their education annually, while in all medical faculties in Croatia in the past 5 years, an average of 700 doctors have graduated annually [20]. At the same time, about 140 doctors annually leave the country [21]. Currently, Croatia is among the three EU countries from which most doctors and nurses emigrate [22].

According to official data, every fourth nurse has emigrated from Croatia to Germany in the last 10 years [23]. According to the HSSMS syndicate [24], Croatia had a shortage of 12,000 nurses even before this epidemic, and according to official statistics, 3180 nurses and technicians went abroad directly from the system from 2013 to 2018 alone, most often to Germany. In the period from 2009 to 2013, 4279 nurses emigrated [25]. The stated data refer only to medical personnel employed in the Croatian health care system, while the number of those who left immediately after finishing school is unknown. So, in the past 10 years alone, 7500 to 8000 nurses have left the health care system in Croatia, making one in four nurses from the system numbering 28,000 [21].

### Emigration of Doctors

According to the Digital Atlas of Croatian Medicine [26], more than 1000 specialist doctors emigrated from Croatia. Another 940 were looking for a letter of resignation. By 2025, the Croatian health care system will have lost another 2700 doctors due to retirement or emigration, according to the estimates by the Croatian Medical Chamber [27]. When considering that in Croatia in 2020 there were 14,094 doctors, these numbers only become alarming in context.

The average age of emigrated doctors from Croatia is 36 years, and the average age of all doctors in the Croatian system is close to 50 years [26]. When considered with how many doctors have emigrated or are preparing for this act in 2020 and 2021 (14%) and the fact that in the next 5 years 2255 doctors will retire (which is 15% of the total number of employees in health care today), it can be concluded that Croatia will lose more than one-third of all doctors in 5 years. Primary health care is the most endangered in terms of staff, and there is already a shortage of 204 family doctors, 75 pediatricians, and 103 gynecologists [23]. There are 2142 family doctors in Croatia, but a third of them are older than 60 years. In Croatian resources, however, no trained staff can replace them [28].

The additional problem is that, due to the decreasing number of children in the family, the circle of care for older adults is decreasing. It will be increasingly difficult for future older people to find someone to provide them with immediate care [29] especially because the average pensioner in Croatia and countries of the WB cannot pay for the various types of services he may need from his current income. All this will lead to the growth of expectations from state care institutions.

In Serbia and B&H, the loss of doctors almost doubled between 2010 and 2017, reaching 14% (B&H total doctors abroad: 1688) and 8% (Serbia total doctors abroad: 3339), respectively, in 2017 [2].

OECD shows that the WB countries have the lowest density of health professionals relative to their populations, while at the same time the number of health graduates in per-capita terms is one of the lowest in Europe [30]. Therefore, a further intensification of outward mobility for this category of workers might be devastating for the region [2]. In addition, the WHO Global Code of Practice on the International Recruitment of Health Personnel [31] deems that the recruitment from health systems affected by shortages of health professionals should be avoided. Despite these warnings and although these trends are against the proclaimed values of the European Union and cohesion policy itself, no concrete measures have been taken at the EU level. Therefore, this paper again points to these issues and, regarding the WHO report [5] about countries with critical health workforce shortages, highlights that these issues are also relevant in European countries. The appeal of the paper is that the list should be updated to include the countries B&H, Serbia, and Croatia.

### The Shortage of Health Care Workers in Germany and Austria

#### Germany

According to projections, the potential labor force in Germany will decrease by 16.2 million workers between 2012 and 2050 for purely demographic reasons [19]. The German generations with the highest birth rates will have left working life around 2035. According to model calculations, the net migration with countries of the European Union will soon drop significantly from the current number to slightly below 300,000 [19]. For the next 36 years, an average of between 276,000 and 491,000 people would have to immigrate from third countries every year to Germany for the labor force potential to remain constant [32]. It seems to be that Croatia and the WB are particularly targeted here. For this reason, in 2020, Germany lifted restrictions on work for people from the WB [33,34].

According to a 2015 study by Bertelsmann Stiftung, in the year 2021, Germany must introduce around 100,000 employees in the field of medical care alone, while it was estimated that as many as 200,000 caregivers would be missing by the year 2021 [32]. In the EU health sector, an increase of 1.8 million jobs is expected by 2025 (an increase of 8.1% compared to the current situation). In the same period, 50% of health professionals in the European Union are expected to retire or leave the health sector, creating 11.6 million jobs, which is more than in any other sector [19]. Between 2000 and 2017, employment in the health sector in the European Union has been rising by 42%, compared with a 15% rise in overall employment [30]. On average, the health sector absorbs about 10% of the workforce in the European Union [30].

As previously shown, Germany and Austria today are undoubtedly addressing their HCW shortages by importing HWs from Croatia and the WB, and this trend became even more noticeable during the COVID-19 pandemic (see Results section).

According to the latest data from the German Employment Service, it is evident that every German federal state has a deficit in occupations in the field of care for older adults. In contrast, when it comes to the need for nurses, there is only one province that does not show a deficit [35]. Germany also shows a substantial need in the older adult care sector. It is estimated that in Germany between 300,000 and 600,000 migrant workers are employed in the care of older adults [18]. These are most often women from Eastern European countries and the WB who are mainly mediated to Germany through private agencies. This form of employment is mostly illegal and is becoming more common in Germany and Austria [36]. Faire Mobilität estimates that between 150,000 and 200,000 illegal workers work in the home care sector in Germany alone [37]. As German society grows older and faster, the number of registered job advertisements in geriatric care has increased 2.5 times in the last 10 years [35].

When it comes to doctors, the German official data show that the immigration of foreign doctors (in the amount of 31,000 doctors per year) has successfully filled the gap until 2013 [38]. The COVID-19 pandemic has made the shortage of doctors evident again in Germany. According to Deutsches Ärzteblatt, there are numerous cases that German doctors had to continue working even when in contact with people positive for SARS-CoV-2 due to shortage of staff [39]. The mismatch between supply and demand will continue to increase during the 2021s, while doctors from the so-called German baby boom generation are retiring, which means a 20% loss of all doctors in Germany. Namely, in 2019, 54.1% of all German doctors were older than 65 years [39].

German official policy makes no secret that the import of HCWs is a matter of primary national importance. Thus, at the end of 2019, a state agency was opened in Saarbrücken to assist in transferring carers and doctors [40]. This state agency intends to speed up immigration procedures with the German authorities for HWs recruited by private employment companies, hospitals, and nursing homes from abroad [40]. The goal is for foreign nurses to immigrate to Germany within 3 months of applying for a visa, a process that lasted up to 2 years until 2019.

#### Austria

In the case of Austria, the inflow of foreign-trained doctors has compensated for 60% of outward mobility, but in the case of nurses, the inflow is outpacing the outflow [7]. However, although in 2010 the number of medical graduates was more than two times higher than in the EU 28 at 22 per 100,000 inhabitants, over the past decade, this ratio has shrunk substantially by one-third to just 14 medical graduates per 100,000 inhabitants—close to the EU 28 average [2]. The number of graduate nurses per capita is below the EU 28 average and has remained unchanged from 2010 to 2017. Consequently, Austria relies much more on nurses originating from other countries, who accounted for a share of 18% as of 2019, than on medical doctors, with a share at 6% as of 2018 [2]. Shortly, the demand for health professionals in Austria is expected to surge rapidly. Close to 30% of doctors in Austria are 55 years and older. According to the European Centre for the Development of Vocational Training, there were more than 13,600 job vacancies for health professionals in Austria at the end of 2019 [2].

Regarding the mentioned WHO report, the paper emphasized that the concept of sustainability of health care systems in the European Union is unsustainable if high-income countries do not train and retain sufficient health workers to meet the need.

### Brain Drain and the Push-and-pull Factors of Health Worker Mobility in the European Union

#### Wage Differential as a Push Factor for East-West Migration

The free movement of workers within the European Union has had an important impact on mobility patterns, especially health workers [41]. The phenomenon is complex, and its drivers are related to economic and institutional factors [8] as well as sociopolitical factors [42].

Expectations about employment opportunities are recognized as important pull factors for the mobility of HCWs. Higher levels of earnings in this sector in a potential host country and relatively high wage differentials between sending and host countries positively impact attracting health professionals in the potential host countries [2]. In 2018, average monthly wages per employee in health work activities in Croatia and the WB were 2 to 3 times lower than in the EU 28 countries [30]. OECD shows that health professionals’ wage differential is a pull factor for east-west migration for this category of workers [30]. The wage differential in the health sector between the EU–Central and Eastern European (CEE) countries and Germany and Austria is substantial and, as such, is an important pull factor of mobility for health professionals from the EU-CEE, Western Balkan countries, and Croatia. Similar patterns and wage gaps apply to nurses [2]. Wage differentials in the health sector across the European countries certainly make some countries more successful at attracting health professionals than other countries that are failing to retain them [2]. Consequently, this group of countries face considerable challenges to provide health assistance to their own rapidly ageing populations, especially in the context of the COVID-19 crisis.

#### Motives of HW Emigration From Croatia

As a reason for their dissatisfaction and motives for emigration from Croatia, nurses express difficult working conditions due to the insufficient number of employees and nonemployment of new nurses [43], inability to advance in the profession according to education and work experience, many unpaid overtime hours, fatigue, and exhaustion [44]. Besides, the perception of corruption in the country, the feeling of legal inequality, and the general negative social atmosphere that prevailed after the exodus of emigration since 2013 also play an essential role in this process [42].

Another issue that needs to be discussed in this section is how the pandemic reflected dissatisfaction with the working conditions of medical staff. The risk of anxiety and other negative mental health reactions among the workforce was described in a viewpoint by Shanafelt et al [45]. The toll of the crisis has been heavy on HCWs [46]. During COVID-19, a higher occurrence was found for all measured negative personal symptoms and negative professional symptoms [47]. The founded association between COVID-19 and mental health was generally the strongest for nurses, age groups 30-49 years, and residential care centers [47]. Prolonged stress at work can lead to burnout syndrome. It is associated with different consequences such as psychosomatic problems, lower employee performance, and more substantial depression and drug consumption. Teachers, police officers, nurses, and doctors have a prevalence in the population between 35% and 40% [48].

Although there are still no studies on this issue in Croatia and the WB, there are numerous testimonies of nurses and doctors in the Croatian media about dissatisfaction with working conditions during the pandemic, manifested in several strikes during the pandemic [49]. According to testimonies, this crisis also contributed to the search for better working conditions through emigration.

#### Consequences of the Pandemic on the Brain Drain From the EU Periphery

Humphries et al [3] illustrated how the pandemic intensified and reinforced, rather than radically altered, the dynamics of doctor emigration in the case of Ireland. According to the authors, the pandemic will likely increase global competition for doctors. The sending countries are at risk of losing out in the game of international health worker recruitment. Hospital doctors must access good working conditions, training, and career progression in the national health systems. Otherwise, the emigration of HWs could threaten the national health system’s capacity to respond to future waves of the pandemic.

The European Committee of the Regions warns that the phenomenon of brain drain poses a risk to the long-term sustainability of the European project if social and economic imbalances between sending and receiving regions remain unaddressed. “It is crucial to achieving a balance between two essential principles of the European Union: free movement of labor and economic and social convergence between regions. Citizens and workers must be able to move freely within the EU, but only because they want to and not because they are pushed from their regions by poverty and scarce economic opportunities” [50].

#### Doctors in Germany From Croatia, Serbia, and B&H

The number of foreign-trained doctors working in OECD countries increased by 50% between 2006 and 2016 (to reach nearly 500,000 in 2016), while the number of foreign-trained nurses increased by 20% over the 5 years from 2011 to 2016 (to reach nearly 550,000) [30] (see Table 2 for Croatia, Serbia, and B&H).

In some countries of the European Union, the percentage of foreign-trained doctors has reached 30% and, in some specializations, has risen to 40%. According to Knez et al [22], both economic and noneconomic factors influence nurses’ and doctors’ choices to emigrate. Benefits of emigration for Croatian nurses and doctors are high satisfaction with living standards, income, professional development, and better work conditions [22].

**Table 2:** Doctors in Germany from Serbia, Croatia, and B&H.^a^

Year	2010, n	2011, n	2012, n	2013, n	2014, n	2015, n	2016, n	2017, n	2018, n	2019, n
Overall number of doctors from B&H^b^ in Germany	118	150	165	202	236	270	327	397	470	505
Annual inflow from B&H	N/A^c^	32	15	37	34	34	57	70	73	35
Overall number of doctors from Serbia in Germany	246	292	381	501	648	826	1026	1236	1364	1504
Annual inflow from Serbia	N/A	46	89	120	147	178	300	210	128	140
Overall number of doctors from Croatia in Germany	137	158	175	196	254	295	341	380	412	428
Annual inflow from Croatia	N/A	21	16	21	63	57	48	46	42	26

^a^Source: Own elaboration from Organisation for Economic Co-operation and Development (2021) [51].

^b^B&H: Bosnia and Herzegovina.

^c^N/A: not applicable.

#### Who Benefits From the Freedom of Movement of Workers in the European Union?

According to Lutz et al [52], the European Union’s future labor force is likely to be smaller but better educated. Simultaneously, overall, in the European Union, the total cost of ageing (public spending on pensions, health care, long-term care, education, and unemployment benefits) is expected to increase by 1.7 percentage points to 26.7% of gross domestic product (GDP) between 2016 and 2070 [52].

**Figure 1:**
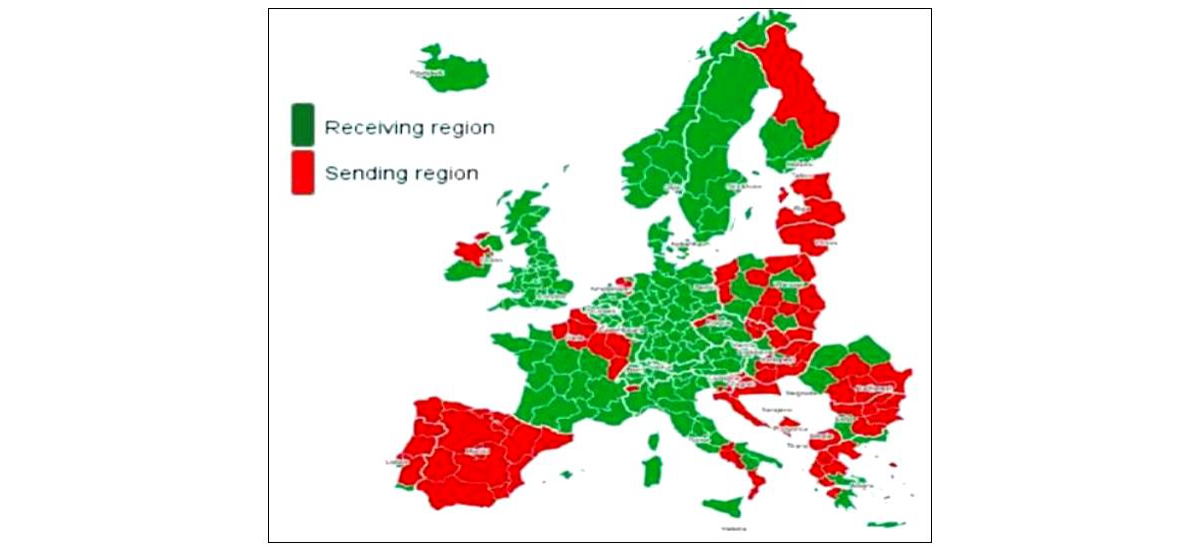
Intra-European Union mobility: receiving and sending countries, nomenclature of territorial units for statistics classification NUTS-2, 2017.

The European Committee of the Regions report shows that intra-EU mobility concerns only a small percentage of Europeans. In 2017, out of a total population of about 511 million citizens, there were almost 17 million EU 28 movers (ie, about 3%) [53]. The ESPON project shows that the sending regions have an average GDP per capita that is 64% of the EU 28 average, while receiving regions have an average GDP per capita of 108% of the EU 28 average. Further, it is indicated that emigration flows follow east-west, south-north, and rural-urban patterns, and that peripheral region are mostly sending regions [54] (see Figure 1).

Freedom of movement of workers in the European Union has doubled since the beginning of the century and is largely beneficial from east to west. Alcidi and Gros [55] examined the factors driving growth in intra-EU labor mobility and showed that just under 4% of EU citizens of working age (20-64 years) now reside in a member state that is not that of their citizenship, ranging from 1.0% of German nationals living and working abroad to 14% of Croatian and 20% of Romanian nationals [55]. Although this mobility is beneficial for receiving states and contributes to a well-functioning monetary union, it can negatively affect the sending countries, resulting in a brain drain and an erosion of public finances [55,56].

## Methods

### Google Trends as a Source of Data for Predicting the Migration of Health Care Workers

Digital footprint monitoring is the primary source of innovation in the context of digital demography [57,58]. Namely, as more and more people are leaving their digital traces on the web, the use of these data for different types of research is becoming more common. In recent years, significant efforts have been made to devise new methods that, in addition to the existing ones, can provide some answers to open questions in the field of demography and public health [57,58].

According to Jurić [59] and Choi and Varian [60], GT data have been used in various types of research: US unemployment, flu outbreak, predicting consumer behavior, predicting inflation rates, predicting the housing market, predicting stock market changes, modeling tourism demand, etc. All the research results showed that the use of GT analytical tools could reveal valuable insights about intentions [61]. Analysis of data obtained through the Google Search engine is therefore gaining in importance in many social sciences [62], and this paper will show that this approach can be beneficial in forecasting the migration of health workers.

The need for such nonstandard approaches to modeling emigration assessments of medical staff is necessary on the one hand due to delays in official data and on the other because there is substantial uncertainty regarding the sustainability of the medical system in connection with the current COVID-19 pandemic. Traditional data sources, based either on surveys or registers, generally fail to provide statistical information on migration flows quickly and do not facilitate correct anticipation of these flows in the short term [63].

Google is the first source of information for most users planning to relocate [64]. Several studies have used immense data sources to analyze migration-related phenomena directly. The first successful analysis of this type of data was in 2009, and the first study in the field of migration examined, during the 2015 migration crisis, searches for particular terms in Arabic in Turkey and Germany according to selected terms such as “Greece” or “Germany” [65]. A study by the Pew Research Center showed that digital prints left by internet searches could provide insight into the movement of migrants. Namely, during their travels in 2015 and 2016, many migrants used smartphones that provided access to information, maps, and travel tips via social media. It was then unequivocally proven that these indicators could be used to predict migration (unpublished study by Jurić). In addition, Böhme et al [66] used a combination of economic and migration-related keywords to predict the levels of migration between groups of countries, with a rather good predictive power.

Compared to approaches using social networks [57,58], the advantage of GT is that limitations related to penetration rates, variable-level social network use, and fake accounts are not prevalent [64]. The main advantage of this approach is the timely detection and identification of external migration, which is an essential analytical indicator for public health, from the labor market to preserving the health system. With this approach, insights into migration trends are obtained a year earlier than official data, which can be used to model projections and predict different trends.

Every year, the global spread of the internet and digital technologies radically transforms the way people communicate with each other, and with the advent of COVID-19, that process has been further accelerated. The COVID-19 outbreak and lockdown accelerated the adoption of digital solutions at an unprecedented pace, creating unforeseen opportunities for scaling up alternative approaches to social science [67]. As a result of the fourth digital revolution and the pandemic, individuals have begun to leave an increasing number of traces online [62]. According to Internet World Stats [68], when it comes to the use of internet services, Croatia, Serbia, and B&H are in the group younger than 50 years, generally comparable to the EU average.

As the internet penetration rate accelerates and increases substantially compared to the creation of credible registration systems for monitoring migration and changes in public health [69], developing tools that retrieve alternative new sources of information is likely to become an accepted additional approach to monitoring demographic trends of all kinds [62]. Although the pandemic accelerated the uptake of digital solutions in data collection techniques [70], the research on the use of substantial data sources (big data) in the field of migration in Southeastern Europe, except our forthcoming study (unpublished study by Jurić), does not exist.

Understanding why health care personnel emigrate from Croatia and the WB, and the consequences of this process are crucial to enabling state agencies and governments to develop optimal intervention strategies to retain this staff and protect the functioning of the health system [71]. For this purpose, we created a method that can be useful for monitoring this process and further predictions of the general interest in emigration.

### Methodological Concept

Internet data (ie, digital traces) could become transformative for demography, especially in migration studies (unpublished study by Jurić). The main advantages of this approach are that those data are easily collected and generated in real time, they are incredibly robust, and they provide a profound insight into the opinions of individuals [72]. This data can be used to gather insights into what was going on in the user’s mind through a noninvasive manner [73]. Moreover, digital traces provide documentation of both movement and activities, which can help researchers bypass possible sources of error in survey data, such as inability to recall and bias. Finally, digital traces can provide access to groups that are difficult to reach or are generally underrepresented by traditional research techniques [74].

The European Commission examined the feasibility of using big data to study demographic issues [57] and concluded similarly to the UN that big data sources do not replace traditional data sources but can complement them, and they can still be used to assess trends. However, these data are also characterized by several shortcomings, as well as data from traditional demographic sources, which we show in the following section.

The primary methodological concept of our approach is to monitor migration-related searches with the analytical tool GT [75]. This tool shows the popularity of a specific term and shows if a trend is rising or falling. GT does not provide information on the actual number of keyword searches. Instead, it standardizes search volume on a scale of 0 to 100 over the period being examined, with higher values indicating the time when the search volume was the greatest, allowing for verifiable metrics (unpublished study by Jurić) [62]. It should be borne in mind, however, that each of these searches was conducted for its reason and does not answer the researchers’ questions, so Googling the term “Germany” is not necessarily an implication that someone wants to move to Germany but may be interested in living conditions, tourist information, or just looking for the German Bundesliga (unpublished study by Jurić) [62]. Therefore, it is essential to choose the correct terms and pay attention to the overall context by interpreting the results.

The Google Search index cannot estimate the exact number of searches, so with the help of this tool, the exact number of emigrants cannot be estimated, but the increase of the trend can be noticed precisely (unpublished study by Jurić) [62,76]. We tested the method in Croatia, B&H, and Serbia by comparing the findings obtained with GT and official statistics. The findings show that the increase in migration-related searches such as “Krankenschwester/ Krankenpfleger + Bewerbung” (Nurse, application for job Germany, Austria) is correlated with increased emigration of HCWs recorded by official statistics and that the decrease in results correlates the decrease of emigrated HWs.

To standardize the data, we requested the data for the period from January 2010 to December 2020 and then divided the keyword frequency for selected words, giving a search frequency index. This index is then compared with official statistics to prove the significance of the results (see further explanations by Wilde et al [77] and Wanner [64]).

Initially, keywords were chosen by brainstorming possible words that we believed to be predictive, specific, and common enough to forecast HCW migration. After the significance screen, we selected the following keywords and topics (Textbox 1).

**Keyword and topic selection criteria.**

**General terms**

- Arbeit (work)
- Job in Austria, Germany
- Gehalt (salary)
- Lohn (salary)
- Job in Germany, Austria
- License

**Job**

- Arbeit + Krankenschwester (work + nurse)
- Arbeit + Arzt (work + doctor)
- Posao + medicinska sestra + Austrija + Njemačka (work + nurse + Austria + Germany)
- Posao + doktor + Austrija + Njemačka (work + doctor + Austria + Germany)

**Application for job**

- Krankenschwester/Krankenpfleger + Bewerbung (nurse, application for job Germany, Austria)
- Arzt + Bewerbung (doctor + application for job Germany + application for job Austria)

### Limitations of the Methodological Concept

Like all others of this type, this study has significant limitations that we want to highlight. Although previous research in this area has shown the feasibility of using digital data for demography, at the same time, we highlight the problems associated with assessments and conclusions (unpublished study by Jurić) [57,62,74,78]. Namely, it is unquestionable that there are still significant open methodological issues with the questionable integrity of the data obtained using the sources of large data sets (unpublished study by Jurić) [62,74].

- The Google Search index cannot estimate the exact number of searches, so with the help of this tool, the exact number of emigrated HCWs cannot be estimated, but the increase of the trend can be noticed precisely, which can serve as an indicator.
- Although the data obtained with GT are robust data with large samples, which provide information qualitatively different from what can be obtained from the official statistics report, Eurostat, OECD, and other official databases, they are not representative of the observed population. It is also a problem that GT does not provide data on which the population was sampled or how it was structured [62].
- A particular problem exists in the researchers’ education, who must be skilled in computational methods, be transparent about their methods to ensure repeatability, and be accustomed to the interdisciplinary environment.
- The last item is both a limitation and an advantage of this approach. Namely, in a traditional research process, a researcher with a predefined theoretical framework and questions collects data from a survey using a carefully crafted set of definitions for each item in the survey. With digital data, the reverse process of operationalizing the research concept occurs. The researcher first observes all the activities and then puts them in a theoretical framework [73].

Unquestionably, this model has unresolved issues related to the reproducibility of the findings and the validity of the measurements, which arise from the characteristics of the GT system used. Although these open-ended issues pose serious challenges for making precise estimates, statistics offer various tools available to deal with imperfect data (unpublished study by Jurić) [62].

## Results

### Use of the Google Trends Analytical Tool to Forecasting the Migration of Health Workers

#### Croatia

**Figure 2:**
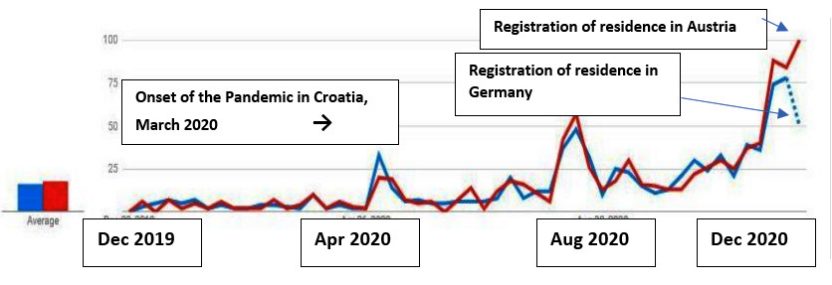
Search queries in German from Croatia "Registration of residence in Germany + PCR" or "Registration of residence in Austria + PCR Test" (searching for a term in Croatian and German from Croatia; 2019-2020). PCR: polymerase chain reaction.

Searching for job applications in Croatia during 2020 (German: “Bewerbung + Krankenschwester”) was more common than the search for the equivalent in Croatian (Croatian: “zamolba za posao + medicinska sestra”). It is also evident that this is an upward trend. This is a strong indication that Croatian citizens continue planning to emigrate to Austria and Germany (unpublished study by Jurić). A further indication is a search for terms related to residence registration in Austria and Germany in combination with the entry “PCR [polymerase chain reaction] test” (Figure 2).

Search queries regarding an application for a job as a nurse in Germany and Austria increased during the 2020 pandemic.

**Figure 3:**
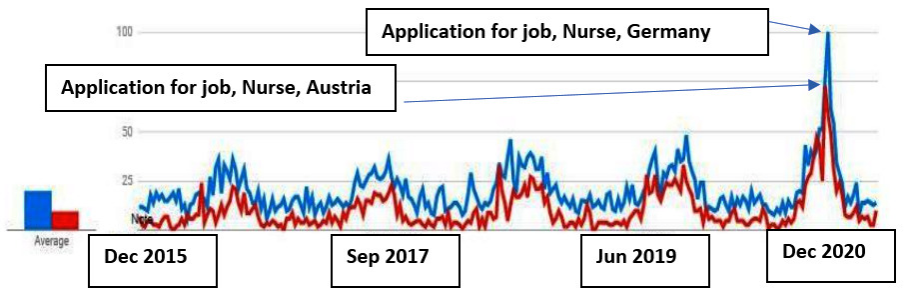
Search queries in German from Croatia "Krankenschwester/Krankenpfleger + Bewerbung Deutschland" and "Austria" (nurse + application for job Germany + application for job Austria; 2015-2020).

Search queries in German from Croatia “Krankenschwester/Krankenpfleger + Bewerbung Deutschland” and “Austria” (nurse + application for job Germany + application for job Austria) increased particularly during the 2020 pandemic (Figure 3). This could be correlated with the difficult working conditions of HCWs in Croatian hospitals during the pandemic [26] but also with the mentioned increased recruitment of HWs from Germany.

**Figure 4:**
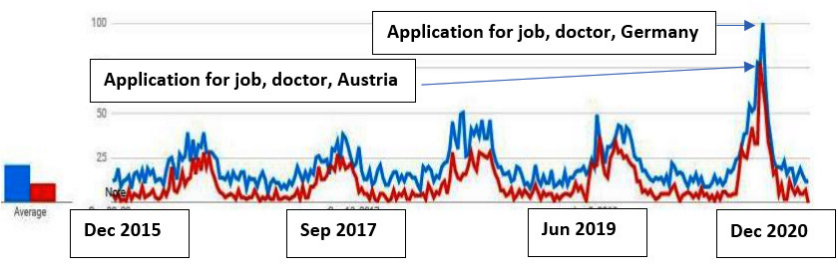
Search queries in German from Croatia "Arzt + Bewerbung, Deutschland" and "Arzt + Bewerbung + Austria" (doctor + application for job Germany + application for job Austria; 2015-2020).

The same phenomenon is also observed in the case of the emigration of doctors (Figure 4).

In further proceedings to standardize the data, we requested the data from January 2010 to December 2020 and divided the keyword frequency for each word and compared this search index with official statistics to prove the significance of results [79].

**Figure 5:**
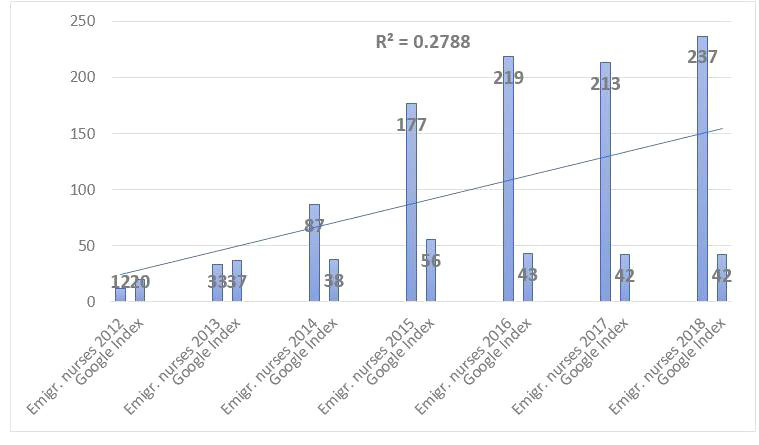
Correlation between Google Search index for query "posao u Njemačkoj + medicinska sestra" (work in Germany + nurses) in Croatian and the Organisation for Economic Co-operation and Development statistics for emigrated nurses from Croatia to Germany (annual inflow). Emgir: emigrate.

Figure 5 shows that the increase in Google Search for the query “posao u Njemačkoj + medicinska sestra” (work in Germany + nurses) correlates with the increase of emigrated nurses to Germany. In the following, we show that the verification can also be performed in the opposite way (ie, from Croatia in German), which again gives reliable estimates.

**Figure 6:**
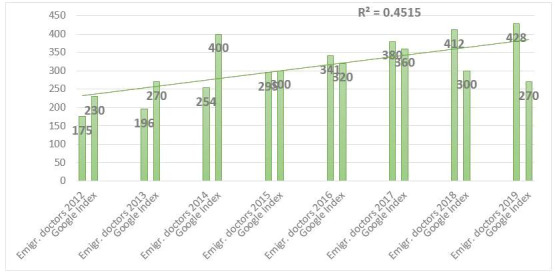
Correlation between Google Search index for query "Arbeit in Deutschland + Arzt" (work in Germany + doctor) in German in Croatia and the Organisation for Economic Co-operation and Development statistics for emigrated doctors from Croatia to Germany. Emgir: emigrate.

Figure 6 shows that, in the case of emigration of doctors, the increase in the Google Search query “Arbeit in Deutschland + Arzt” (work in Germany + doctor) was correlated with the increase of emigrated doctors to Germany.

#### B&H and Serbia

**Figure 7:**
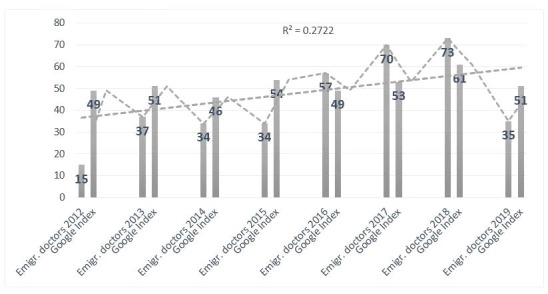
Correlation between Google Search index for query "Arbeit in Deutschland + Arzt" (work in Germany + doctor) in Bosnia and Herzegovina (B&H) and the Organisation for Economic Co-operation and Development statistics for emigrated doctors from B&H to Germany (annual inflow). Emgir: emigrate.

In the case of B&H, we calculated the annual inflow of B&H doctors to Germany and compared these data with the GT index (Figure 7). As in the case of Croatia, there was a positive correlation.

**Figure 8:**
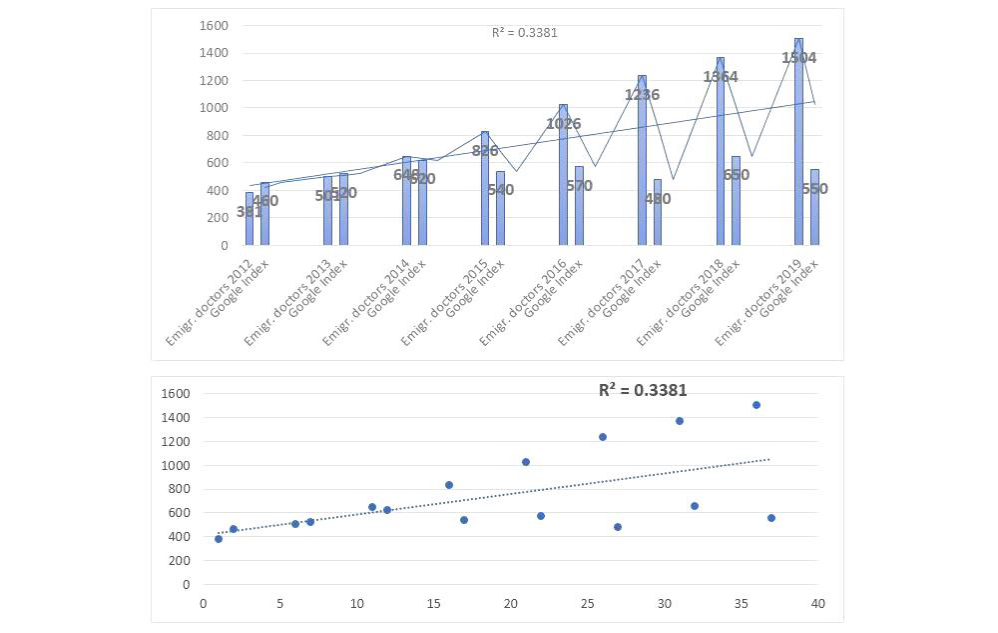
Correlation between Google Search index for query "Arbeit in Deutschland + Arzt" (work in Germany + doctor) in Serbia and the Organisation for Economic Co-operation and Development statistics for emigrated doctors from Serbia to Germany. Emgir: emigrate.

In addition, in the case of Serbia (Figure 8), the increase in Google Search for the query “posao u Nemačkoj + Doktor” (work in Germany + doctor) correlated with the increase of emigrated doctors to Germany. There is a positive linear association between the Google index and data from official statistics (OECD).

## Discussion

From 2010 to 2020, 65,288 HWs emigrated from Croatia and the WB. Without such intense emigration in the last 10 years, Croatia and the WB would have 50% more HWCs today. It is necessary to emphasize that this staff is crucial in the fight against a pandemic.

During the pandemic, the “normal” ways of data collection are simply too slow (particularly when EU countries are fast tracking health workers into the European Union). The methods presented here show a way of generating timely insights into intent to migrate among health workers. All tested migration-related search queries that show an indication about HCWs’ emigration planning showed a positive linear association between Google index and data from official statistics (OECD: Serbia *R*^2^=0.3381, B&H *R*^2^=0.2722, Croatia *R*^2^=0.4515). The increase in Google Search is correlated with the increase in the number of emigrated HWs from Croatia, Serbia, and B&H. The decrease in Google Search is correlated with the decrease in the emigration of HWs.

This method contributes in a way that proves the feasibility of predicting further migrations from Croatia, Serbia, and B&H in this specific case of HCWs to Germany and Austria, which allows reliable forecasts for the future. This procedure also presents a new methodological approach to how data obtained through GT can be standardized for comparison with official databases.

The insights are particularly relevant for national and EU policy makers, and can help design appropriate strategies to retain HCWs. The method can enable state agencies and the government to prepare and better respond to the shortage of HWs in the future and protect the functioning of the health system. Regarding the WHO report about countries with critical health workforce shortages, this paper highlights that these issues are also relevant in European countries and that the list should be updated to include the countries B&H, Serbia, and Croatia. In addition, it is emphasized that the concept of health care system sustainability in the European Union is unsustainable if high-income countries do not train and retain sufficient health workers to meet the need.

Although this mobility is beneficial for receiving states and contributes to a well-functioning monetary union, it negatively affects the sending countries, resulting in a brain drain and an erosion of public finances [55]. The issue of the European Union drawing HWs from the EU periphery (Croatia) and nearby countries (B&H, Serbia) clearly shows a clash between the EU free movement and the right to health care and a need to ensure a health workforce in all European regions (as per the WHO global code and the UN Sustainable Development Goals).

This method could be useful for policy makers but only if they respond and react to the data. An important question for policy makers is how they can retain health workers during a pandemic. Increased salaries and improved working conditions is certainly a good way. What precisely could the European Union do to address this problem? One approach would be to strengthen fiscal transfers to the member states and countries of the European periphery that are most affected by the harmful effects of freedom of movement [56]. However, fiscal transfers can never fully compensate for the loss of population. For example, financial compensation cannot fully compensate the departure of a nurse who left a Croatian hospital and now works in Germany until a Croatian hospital finds a replacement. Otherwise, the specific hospital will still lack a nurse, which is reflected in Croatia’s general quality of health care. That is why we proposed a compensation solution so that Germany funds centers of excellence for the education of nurses in Croatia and the WB, if they remain to work in their homeland for 5 years after completing their education. In this context, it welcomes the appeal of the WHO that calls on high-income countries to strive for self-sufficiency through educating, retaining, and sustaining enough doctors and nurses to staff their health care systems [22].

In a situation where there is only freedom of movement of workers but not a common pension and health care system in the European Union, or a guaranteed minimum wage, nothing significant will change at the EU level. This means that the EU framework remains a structure in which the wealthy members will continue to become richer and the poor members increasingly poorer, which also applies to the whole European periphery. Moreover, with the onset of the pandemic, the situation worsened.

Without systemic regulation of this issue at the EU level, such trends of the emigration of HWs will threaten the national health system’s capacity to respond to the needs of an ageing population and possible new waves of the pandemic.

## Abbreviations

B&H: Bosnia and Herzegovina
CEE: Central and Eastern European
GDP: gross domestic product
GT: Google Trends
HW: health care worker
OECD: Organisation for Economic Co-operation and Development
PCR: polymerase chain reaction
UN: United Nations
WB: Western Balkans
WHO: World Health Organization
